# Hematological Profiles and Oxidant–Antioxidant Status in Native Turkish White Geese: A Preliminary Comparison Between Young and Adult Geese of Both Sexes

**DOI:** 10.3390/vetsci13090935

**Published:** 2026-09-09

**Authors:** Elmas Ulutaş, Çağatay Salum, Ahmet Araman, Erkan Çalık, Mehmet Akif Boz

**Affiliations:** 1 Department of Physiology, Faculty of Veterinary Medicine, Yozgat Bozok University, Yozgat 66900, Türkiye; 2 Department of Physiology, Faculty of Veterinary Medicine, Kastamonu University, Kastamonu 37150, Türkiye; csalum@kastamonu.edu.tr; 3 Institute of Health Sciences Master’s Program, Veterinary Internal Medicine, Afyon Kocatepe University, Afyonkarahisar 03200, Türkiye; ahmetaraman881@gmail.com; 4 Private Veterinary Clinic, Afyonkarahisar 03100, Türkiye; vet.erkancalik@gmail.com; 5 Department of Animal Science, Faculty of Agriculture, Yozgat Bozok University, Yozgat 66900, Türkiye; m.akif.boz@bozok.edu.tr

**Keywords:** geese, hematological profile, oxidant–antioxidant status, age and sex

## Abstract

Hematological parameters and indicators of oxidant–antioxidant balance could provide useful information about the physiological characteristics of poultry. However, information on these parameters in native Turkish white geese of different ages and sexes remains limited. This study evaluated 40 clinically healthy native Turkish white geese aged 16 or 98 weeks (10 females and 10 males per group). Hematology was assessed manually; plasma total antioxidant status (TAS), total oxidant status (TOS), and oxidative stress index (OSI) were measured with commercial kits. Two-way ANOVA was followed by Bonferroni-adjusted comparisons where appropriate. To aid the interpretation of the statistical findings, effect sizes for the main effects of age and sex, as well as for the age × sex interaction, were expressed as partial eta-squared (ηp^2^) and reported with 95% confidence intervals. The age × sex interaction was significant for erythrocyte count, packed cell volume (PCV), mean corpuscular hemoglobin concentration (MCHC), and all leukocyte parameters except basophil percentage. Young males had higher erythrocyte counts and lower MCHC than adult males, whereas young and adult females had similar values. PCV was higher in young than adult geese within both sexes. Within both sexes, adults had higher total leukocyte counts, heterophil and eosinophil percentages, and heterophil-to-lymphocyte (H/L) ratios, but lower lymphocyte and monocyte percentages. No significant interaction was detected for TAS, TOS, or OSI; TAS was higher in adults, whereas TOS and OSI were higher in females. These results provide comparative data for interpreting hematological and oxidant–antioxidant measurements in native Turkish white geese at two distinct ages under similar rearing conditions.

## Simple Summary

Blood tests and indicators of oxidant–antioxidant balance could provide useful information about the physiological characteristics of poultry. However, information on these parameters in native Turkish white geese of different ages and sexes remains limited. In this study, hematological parameters and oxidant–antioxidant status were compared among young and adult female and male native Turkish white geese. Some hematological parameters differed according to age and sex. Moreover, total antioxidant status differed between young and adult geese, whereas total oxidant status and the oxidative stress index differed between females and males. Significant age × sex interactions were detected for several hematological parameters. In contrast, no significant age × sex interaction was detected for total antioxidant status, total oxidant status, or the oxidative stress index. These findings provide preliminary information on physiological variation in clinically healthy native Turkish white geese and may assist the interpretation of these parameters in future studies of this native genetic resource.

## 1. Introduction

Geese are an important poultry species raised worldwide for meat, liver, feathers and egg production [1]. Their ability to efficiently utilize high-fiber feeds and pasture resources, adapt to free-range production systems, withstand harsh climatic conditions, exhibit relative resistance to diseases and require minimal housing makes them particularly suitable for extensive production systems [2,3,4,5]. Goose production in Turkey is primarily based on traditional family farming under extensive conditions and continues to hold economic and cultural significance due to its contribution to household nutrition and the rural economy [6]. Despite these production and adaptation advantages, scientific knowledge regarding the physiological characteristics of native Turkish geese, particularly their hematological parameters and oxidant–antioxidant status, remains limited [7,8,9,10].

Hematological parameters and indicators of oxidant–antioxidant balance could provide useful information about the physiological characteristics of birds. Erythrocyte-related parameters provide information on erythrocyte mass and oxygen-carrying capacity, whereas leukocyte profiles may reflect aspects of immune and physiological responses [11,12]. The heterophil-to-lymphocyte (H/L) ratio is widely used as a potential stress-related parameter in birds; however, it is not specific to stress and may also vary with age, season, reproductive status, environmental conditions and other physiological factors [12]. Since the erythrocytes and platelets of avians are nucleated, the automated cell counting methods commonly used in mammals may not be reliable. For this reason, manual hemocytometer counting and microscopic leukocyte differential counts remain important in avian hematology [11,13]. Total antioxidant status (TAS), total oxidant status (TOS), and the oxidative stress index (OSI) provide information on antioxidant capacity, overall oxidant status and the balance between oxidant and antioxidant systems, respectively [14]. Therefore, the combined evaluation of hematological parameters and oxidant–antioxidant status may provide complementary information about physiological variation in birds.

Hematological parameters in birds may vary depending on various biological and environmental factors, such as age, sex, diet, environmental conditions and management practices [10,15,16]. Consequently, studies on geese have reported inconsistent findings regarding age and sex-related variations in hematological parameters. Thavasiappan et al. (2023) reported higher erythrocyte and hemoglobin values in female geese; Hamadani et al. (2014) and Jahantigh and Zamani-Ahmadmamudi (2016) found no significant sex-related differences in these parameters [8,9,10]. Similar inconsistencies have been reported for other hematological parameters, suggesting that differences in breed, physiological stage, diet, rearing conditions and study design may contribute to the variations observed across studies [7,17]. However, studies simultaneously evaluating the effects of age, sex, and their interaction on hematological parameters and oxidant–antioxidant status in geese under standardized management conditions remain limited.

Although previous studies on native Turkish geese have provided information on production performance, growth, carcass characteristics, reproduction, incubation characteristics, egg quality, meat quality and fatty acid composition [4,18,19,20,21], relatively less attention has been paid to their physiological characteristics. Among the limited physiological studies, oxidant–antioxidant parameters have been investigated in relation to live weight and sex; however, hematological parameters were not evaluated concurrently, and age-related differences were not assessed [22]. Therefore, information obtained through the simultaneous evaluation of age, sex, hematological parameters, and oxidant–antioxidant status in native Turkish white geese remains limited. Accordingly, the present study aimed to evaluate hematological parameters and oxidant–antioxidant status in clinically healthy native Turkish white geese and to determine the effects of age, sex, and their interaction on these variables under standardized management conditions.

## 2. Materials and Methods

### 2.1. Ethics Statement

All experimental procedures were conducted in accordance with national and institutional guidelines for the care and use of laboratory animals and were approved by the Animal Experiments Local Ethics Committee of Erciyes University (Kayseri, Türkiye; Approval No: 2021/237, Date: 1 December 2021). The experimental protocol was reviewed and approved prior to the commencement of the study.

### 2.2. Animals, Management and Diet

**Figure 1:**
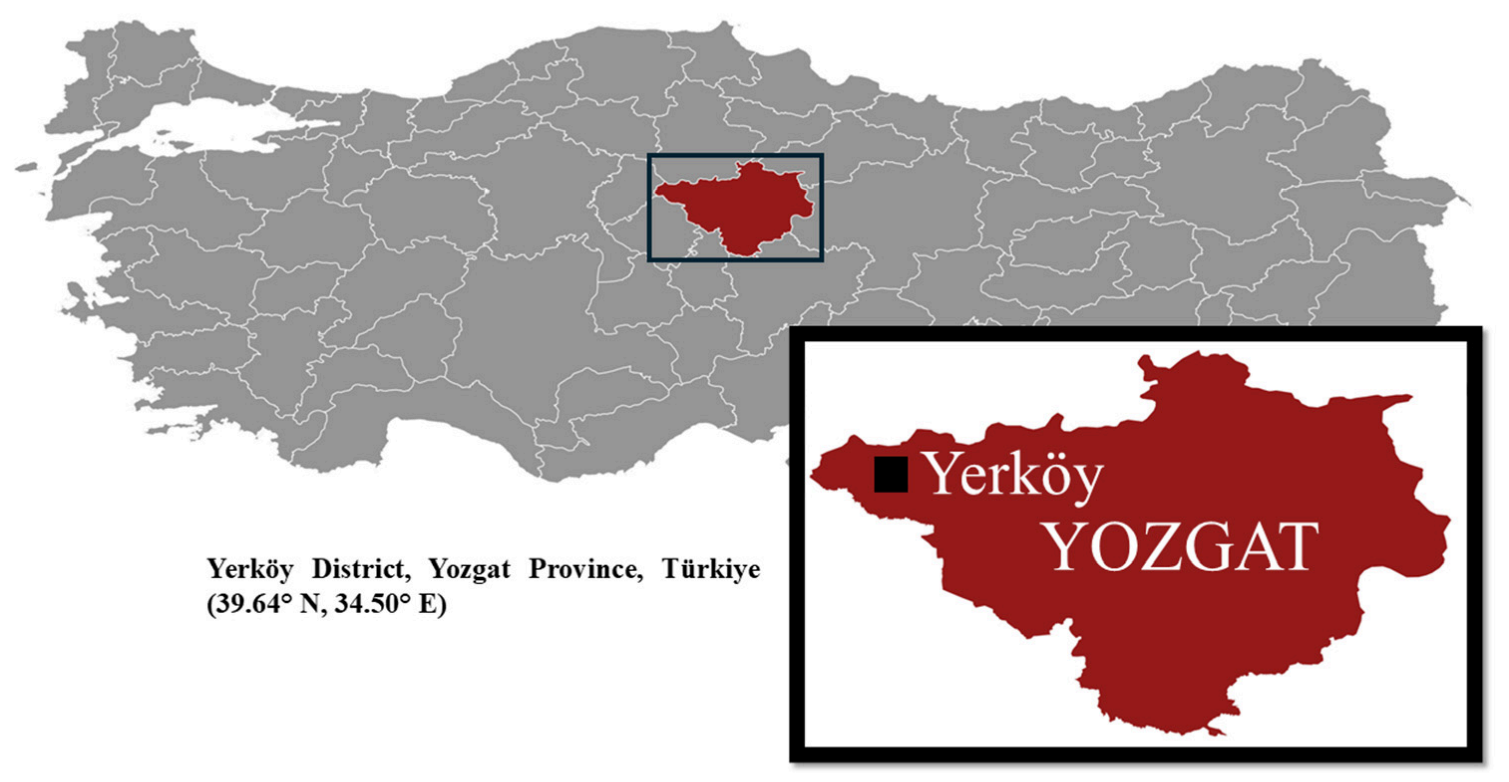
Location and GPS coordinates of the Yozgat Bozok University Agricultural Application and Research Center where the study was conducted.

The study population comprised approximately 300 native Turkish white geese maintained at the Yozgat Bozok University Agricultural Application and Research Center in Yerköy, Yozgat, Türkiye (39.64° N, 34.50° E), a region characterized by a continental climate. The geographical location of the study area is presented in Figure 1. The sample size was determined using G*Power version 3.1.9.7. Analyses were conducted on four groups, and the “ANOVA: fixed-effect, omnibus, one-way” model was used. The calculations used an effect size (f) of 0.60, a significance level (α) of 0.05, a test power (1 − β) of 0.80. The analysis determined that a minimum of 9 animals was sufficient for each group. Actual power was calculated to be 82%. Therefore, 40 geese were used as test subjects in the study. A total of 40 clinically healthy geese were randomly selected from the study population, including 20 young geese (16 weeks of age; 10 females and 10 males) and 20 adult geese (98 weeks of age; 10 females and 10 males).

The geese were housed in a 300 m^2^ indoor shelter with access to a 2400 m^2^ outdoor area, providing approximately 1 m^2^ of indoor space and 8 m^2^ of outdoor space per goose. The outdoor area had a soil-covered surface without established vegetation and was used primarily as an outdoor exercise area rather than for grazing. From 10 weeks of age onward, the young and adult geese were maintained together under the same housing and management conditions and had unrestricted daytime access to the outdoor area. The flock was monitored weekly for signs of disease, injury, or other health problems, and only clinically healthy geese were included in the study. All animals originated from the same native Turkish white goose flock, in which individual pedigree records were not maintained. No vaccination program was applied to the flock. Historical individual records regarding previous reproductive history and parasite-control treatments were unavailable.

As part of the routine feeding program of the native Turkish goose flock, all geese were fed the same starter feed from hatching to 6 weeks of age and the same grower feed from 6 to 10 weeks of age. From 10 weeks of age onward, both young and adult geese were fed the diet described below. The diet contained 15% crude protein, 12.56 MJ/kg metabolizable energy (ME), 5.4% crude ash, 4.2% crude fiber, 0.290% methionine, 0.670% lysine, 0.750% calcium and 0.320% phosphorus. Water was provided *ad libitum*. The geese were exposed only to the natural photoperiod to minimize the potential influence of reproductive status on hematological and oxidant–antioxidant parameters. Blood samples from adult female geese were collected outside of the egg-laying season.

The average body weights of the geese included in the study were determined to be 3680 ± 110 g for young females, 4250 ± 142 g for young males, 4575 ± 145 g for adult females and 5214 ± 185 g for adult males.

### 2.3. Analysis

Blood samples were collected from the brachial (wing) vein of each goose on the same day, between 8:00 and 10:00. Approximately 4 mL of blood was collected into Dipotassium Ethylenediaminetetraacetic Acid (K_2_-EDTA) vacuum blood collection tubes (BD Vacutainer^®^, Becton, Dickinson and Company, Franklin Lakes, NJ, USA) [11,23]. K_2_EDTA tubes were selected because hematological analysis was a primary component of the study. The samples were transported to the laboratory in a cooled container and subjected to immediate hematological analysis. After the required aliquot had been used for hematological measurements, the remaining blood was centrifuged at 635× *g* for 5 min in a refrigerated centrifuge (Universal 320R, Andreas Hettich GmbH, Tuttlingen, Germany). Plasma was carefully separated and stored at −18 °C for approximately one month until TAS and TOS analyses. According to the manufacturer’s instructions, the commercial TAS and TOS assays were suitable for both serum and plasma samples. Therefore, using the plasma remaining after hematological analysis avoided the need to collect an additional blood sample for serum preparation. To minimize the potential effects of freeze–thaw cycles on plasma stability, the plasma samples were thawed only once, immediately before analysis [24].

**Figure 2:**
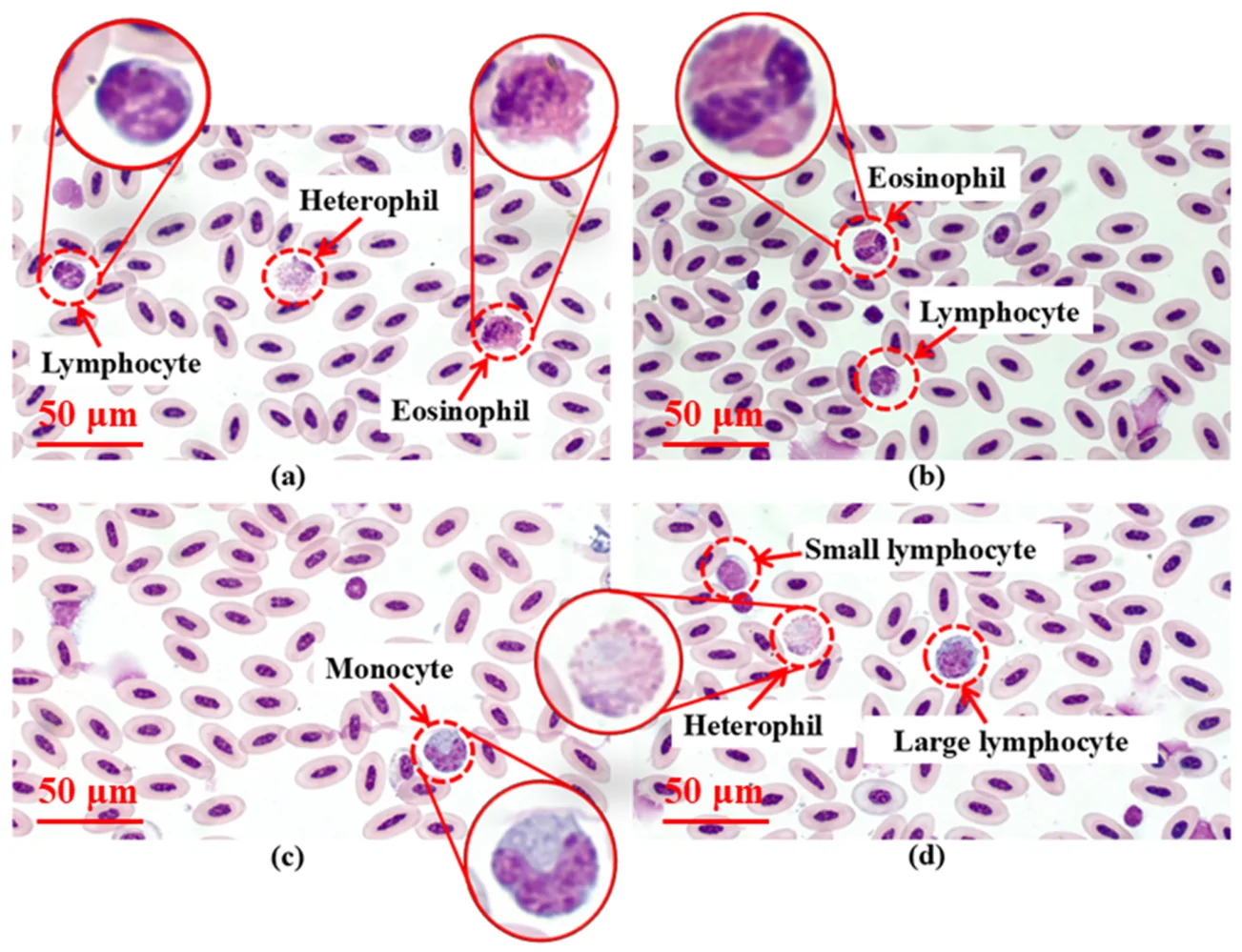
Photomicrographs of May–Grünwald–Giemsa-stained peripheral blood smears illustrating the morphology of leukocyte types identified in native Turkish white geese. (**a**) Lymphocyte, heterophil and eosinophil for young female; (**b**) eosinophil and lymphocyte for young male; (**c**) monocyte for adult female; and (**d**) heterophil, small lymphocyte and large lymphocyte for adult male geese.

Packed cell volume (PCV) was determined by the microhematocrit method using a microhematocrit centrifuge (Hematocrit 200, Andreas Hettich GmbH, Tuttlingen, Germany) [25]. The erythrocyte and total leukocyte counts were determined using a Neubauer hemocytometer (Paul Marienfeld GmbH & Co. KG, Lauda-Königshofen, Germany) with Natt and Herrick’s solution [26]. Mean corpuscular volume (MCV), mean corpuscular hemoglobin (MCH) and mean corpuscular hemoglobin concentration (MCHC) were calculated using standard formulas [27]. Hemoglobin concentration was determined by the cyanmethemoglobin (Drabkin’s) method using Drabkin’s reagent (ChemBio Laboratory Research, Istanbul, Türkiye) and a microplate spectrophotometer (SPECTROstar Nano, BMG LABTECH, Ortenberg, Germany) [28,29]. Two blood smears were prepared from each blood sample and stained using the May–Grünwald (Merck KGaA, Darmstadt, Germany) and Giemsa (Merck KGaA, Darmstadt, Germany) staining method. Differential leukocyte counts were performed by examining May–Grünwald–Giemsa-stained blood smears under a light microscope (Primostar, Carl Zeiss AG, Oberkochen, Germany) using a 100× oil-immersion objective. Leukocyte percentages were determined by counting 100 leukocytes per smear [11]. The images of the blood smears were captured using an Axiolab 5 light microscope (Carl Zeiss AG, Oberkochen, Germany) equipped with an Axiocam 208 color camera. Representative images of the different leukocyte types identified in the present study are shown in Figure 2. Microscopic analyses were performed by two veterinary physiologists. Total erythrocyte and total leukocyte counts were performed twice for each sample. Two blood smears were created and all leukocyte types were analyzed blindly.

TAS and TOS concentrations were determined using commercially available assay kits (Rel Assay Diagnostics, Gaziantep, Türkiye) according to the manufacturer’s instructions and measured using a microplate spectrophotometer (SPECTROstar Nano, BMG LABTECH, Ortenberg, Germany). TAS and TOS concentrations were expressed as mmol Trolox equivalent/L and μmol H_2_O_2_ equivalent/L, respectively, as described by Erel [30,31]. The oxidative stress index (OSI) was calculated using the following Equation (1) [32]:

(1) $$OSI\;(arbitrary\;units)=\frac{TOS\;(\mu mol\;H_{2}O_{2}\;equivalent/L)}{TAS\;(mmol\;Trolox\;equivalent/L)\times 10}$$

### 2.4. Statistical Analysis

Statistical analyses were performed using IBM SPSS Statistics version 27.0 (IBM Corp., Armonk, NY, USA). The effects of age, sex and their interaction on the study parameters were evaluated using two-way analysis of variance (ANOVA). Age consisted of two levels (young and adult) and sex consisted of two levels (female and male); the model was a 2 × 2 factorial design. The normality of the model residuals was assessed using the Shapiro–Wilk test and the homogeneity of variances was assessed using Levene’s test. For parameters where the age × sex interaction was significant, the main effects of age and sex were not interpreted independently. Instead, Bonferroni-adjusted simple effects analyses were performed to compare young and adult geese within each sex and females and males within each age group. Also, Bonferroni-adjusted pairwise comparisons of age × sex means were used to determine differences between groups. Bonferroni-adjusted *p*-values below 0.05 were considered statistically significant. For all other statistical tests, significance was also accepted at *p* < 0.05. Data are presented as mean ± standard deviation (SD). The effect sizes and partial eta-squared (ηp^2^) values for the main effects of age, sex and their interactions were calculated for a 95% confidence interval to inform the interpretation of the statistical data.

## 3. Results

The mean values of hematological parameters and oxidant–antioxidant status in geese according to age, sex, and their interaction are presented in Table 1.

Age and the age × sex interactions were statistically significant for erythrocyte count (*p* < 0.001), whereas the effect of sex was not significant (*p* > 0.05). Young male geese had a higher erythrocyte count than adult male geese, while young and adult female geese had similar values. Age and the age × sex interactions were statistically significant for PCV (*p* < 0.001), whereas the effect of sex was not significant (*p* > 0.05). PCV values were higher in young geese than in adult geese within both sexes. Age was significant for MCHC (*p* < 0.01). The age × sex interaction was also significant (*p* < 0.05), whereas sex was not significant (*p* > 0.05). Adult female and adult male geese had higher MCHC values than young male geese. Young female geese had values similar to those of the other groups. Age, sex, and the age × sex interactions were not significant for hemoglobin concentration, MCV, and MCH (*p* > 0.05).

Age and the age × sex interactions were significant for total leukocyte count (*p* < 0.001), whereas sex was not significant (*p* > 0.05). Adult female and adult male geese had higher total leukocyte counts than young female and young male geese. Age and the age × sex interactions were significant for lymphocyte percentage (*p* < 0.001); however, sex was not significant (*p* > 0.05). Young female and young male geese had higher lymphocyte percentages than adult female and adult male geese. Age and the age × sex interactions were also significant for heterophil percentage (*p* < 0.001), but sex was not significant (*p* > 0.05). Young female and young male geese had lower heterophil percentages than adult female and adult male geese. Age and the age × sex interactions were significant for monocyte percentage (*p* < 0.001) and sex was also significant (*p* < 0.01). The young female group had the highest mean monocyte percentage, whereas the adult male group had the lowest. Young male geese had values similar to those of young female and adult female geese. Age and the age × sex interactions were significant for eosinophil percentage (*p* < 0.001), and sex was also significant (*p* < 0.05). Adult male geese had a higher eosinophil percentage than young female and young male geese, while adult female geese had a higher eosinophil percentage than young female geese. The values were similar between young females and young males, between adult females and young males, and between adult females and adult males. Age, sex, and the age × sex interactions were not significant for basophil percentage (*p* > 0.05). Age and the age × sex interactions were significant for H/L (*p* < 0.001) but sex was not significant (*p* > 0.05). Young female and young male geese had similar H/L values, as did adult female and adult male geese. The adult groups had higher H/L values than the young groups.

When the oxidant–antioxidant status parameters were evaluated, age was significant for TAS (*p* < 0.05), whereas sex and the age × sex interaction were not significant (*p* > 0.05). The TAS value for the young groups was lower than that for the adult groups. For TOS, sex was significant (*p* < 0.01), whereas age and the age × sex interaction were not significant (*p* > 0.05). The young and adult female groups had similar TOS values, as did the young and adult male groups. The female groups had higher TOS values than the male groups. Similarly, sex was significant for OSI (*p* < 0.01); however, age and the age × sex interaction were not significant (*p* > 0.05). The young and adult female groups had similar OSI values, as did the young and adult male groups. The female groups had higher OSI values than the male groups.

The effect sizes of age, sex, and the age × sex interaction on hematological parameters and oxidant–antioxidant status in geese are presented in Table 2. Effect sizes and their 95% confidence intervals provided additional information on the magnitude and precision of the observed effects. Among the oxidant–antioxidant parameters, OSI (ηp^2^ = 0.208, 95% CI: 0.026–0.423) and TOS (ηp^2^ = 0.175, 95% CI: 0.013–0.391) showed the largest sex-related effect sizes. For the age × sex interaction, the largest effect sizes were observed for lymphocyte percentage (ηp^2^ = 0.512, 95% CI: 0.276–0.669), heterophil percentage (ηp^2^ = 0.423, 95% CI: 0.181–0.603), H/L ratio (ηp^2^ = 0.392, 95% CI: 0.152–0.580), erythrocyte count (ηp^2^ = 0.372, 95% CI: 0.134–0.564), and monocyte percentage (ηp^2^ = 0.347, 95% CI: 0.113–0.544).

## 4. Discussion

In this study, the hematological profiles and oxidant–antioxidant status of clinically healthy native Turkish white geese aged 16 and 98 weeks were evaluated in relation to age, sex, and the age × sex interaction. Among the erythrocyte parameters, significant age × sex interactions were observed for erythrocyte count, PCV, and MCHC. Neither age, sex, nor the age × sex interaction was significant for hemoglobin concentration, MCV, or MCH. The observed group differences varied among parameters. Differences between the 16- and 98-week-old groups in erythrocyte count and MCHC were observed only in males; PCV was higher in 16-week-old than in 98-week-old geese within both sexes. Therefore, comparisons between the two age groups were interpreted separately for each erythrocyte parameter and sex.

It has been reported that in poultry, erythrocyte count, PCV, and hemoglobin concentration could be influenced by age, sex, hormonal status, and other physiological factors; erythropoiesis, on the other hand, is regulated by erythropoietin, which is synthesized in the kidneys [33]. The higher erythrocyte count in young males compared to adult males may be related to differences in erythropoietic activity between the two age groups. However, the fact that the same difference is not observed in females indicates that a general physiological explanation based exclusively on age is insufficient. Since erythropoietin, reticulocyte count, iron metabolism, and related hormones were not evaluated, the mechanism underlying this finding specific to males could not be determined with the available data. Thavasiappan et al. (2023) reported that in female geese, erythrocyte count, hemoglobin concentration, and PCV values were higher than those of males only in the 1–2 months-old and 2–5-year-old age groups, while no differences were found between the sexes in other age groups [10]. However, there are also studies that have evaluated the effects of sex on erythrocyte parameters differently. In a study on the adult domestic geese, no significant sex-related differences were found in erythrocyte count, PCV, hemoglobin concentration, MCV, MCH, or MCHC [8]. Similarly, erythrocyte count, PCV, and hemoglobin concentration did not differ significantly between adult female and male greylag geese [9]. Furthermore, it has been reported that PCV is higher in adult wild Canada geese than in young geese and increases with body weight in adults [17]. This finding is contrary to the results of our study, in which PCV values in 16-week-old geese of both sexes were higher than those in 98-week-old geese. Differences between studies may be related to age classification, population characteristics, body weight, husbandry conditions, and the sampling period. Because the differences in erythrocyte count, PCV, and MCHC were not accompanied by significant differences in hemoglobin concentration, MCV, or MCH, the present data do not establish altered oxygen-carrying capacity or increased or decreased erythropoietic activity.

The effect size values for the erythrocyte parameters were examined (Table 2). It was found that age and the age × sex interaction had a greater effect on changes in erythrocyte count. Changes in PCV were also influenced by age and the age × sex interaction. The effect size values for the other erythrocyte parameters were low for the 95% confidence interval.

In the context of the age × sex interaction, the higher H/L ratio observed in both sexes among 98-week-old geese was accompanied by higher heterophil and lower lymphocyte percentages. Since differential leukocyte counts exhibit relative percentages, a lower lymphocyte percentage does not necessarily indicate a decrease in absolute lymphocyte count or suppressed immunity [34]. During the stress response, the redistribution of lymphocytes from the circulation to other tissues and an increase in circulating heterophils may alter the percentages of heterophils and lymphocytes [12]. However, this mechanism’s contribution to the differences observed in our study could not be determined, as absolute differential leukocyte counts and immune function were not evaluated.

The H/L ratio and circulating corticosterone represent different components of the stress response and follow different time courses; therefore, they could not be used inter-changeably [34]. Leukocyte profiles in healthy populations could also vary with age, sex, and season [34]. In a study conducted by Charles-Smith et al. on wild Canada geese, blood samples collected during the annual molting period revealed that adult geese had higher estimated total leukocyte counts, heterophil percentages, and H/L ratios than juveniles, while their lymphocyte percentages were lower [17]. These results are consistent with the differences observed between the two age groups in our study. Whereas the estimated total white blood cell count in females was higher than that in males in the same study, in our study, no significant differences were found between females and males of the same age regarding these four parameters [17]. Studies performed on adult domestic geese and adult greylag geese have also reported no sex differences in total leukocyte count or differential leukocyte percentages [8,9]. However, in wild greylag geese, it has been reported that leukocyte counts may vary more in relation to season, pair-bond status, and rearing conditions than to age or sex [35]. Differences in population, age classification, rearing conditions, and sampling periods limit the direct comparability of these studies. Furthermore, it has been reported that the relationship between the H/L ratio and pair-bond status in greylag geese varies by season [35], while in black-legged kittiwake, the relationship between the H/L ratio and body condition differs across age groups [36]. These results suggest that the H/L ratio may vary not only with age but also depending on social and environmental conditions. Therefore, it could not be determined from the current data whether the higher H/L ratio observed in 98-week-old geese is associated with increased physiological stress.

In both sexes, the percentage of monocytes in 98-week-old geese was lower than in 16-week-old geese. The lowest value was observed in 98-week-old males. Monocytes are circulating cells that play a role in phagocytosis and differentiate into macrophages after migrating to tissues [33]. Accordingly, trafficking of monocytes between blood and tissues could contribute to variation in peripheral monocyte percentages. However, because tissue migration and macrophage activity were not assessed, the contribution of this process to the observed age × sex interaction, including the particularly low value in 98-week-old males, could not be determined. Similarly, previous studies on birds have not shown a consistent trend of change in peripheral blood monocytes with age. Howlett et al. (2002) reported higher lymphocyte and monocyte counts in juvenile bustards than adult values [37]. Furthermore, while relative monocyte counts in young Canada geese were correlated with body weight [17], the percentage of monocytes in free-ranging greylag geese was not associated with age or sex but did vary seasonally [35]. These findings indicate that circulating monocyte levels may vary not only with age but also with species, body characteristics, and environmental conditions.

The precise function of avian eosinophils has not yet been fully elucidated. Although a limited association between eosinophils and nematode infections has been reported in some avian species, parasite antigens generally do not induce eosinophilia in birds [33]. Avian basophil granules contain histamine, and these cells are thought to participate in the initial stages of acute inflammation; however, acute inflammation does not always result in peripheral basophilia [33]. In the present study, the age × sex interaction was significant for eosinophil percentage; within both sexes, 98-week-old geese had higher eosinophil percentages than 16-week-old geese. By contrast, basophil percentage did not differ according to age, sex, or the age × sex interaction. Relative eosinophil percentage was reported to increase as body weight decreased in adult Canada geese [17]. Benarrós et al. (2020) identified eosinophils as the predominant leukocyte type in adult male Chinese geese; however, because goose-specific reference data were limited, they did not interpret this finding as a pathological change and did not exclude parasitism [7]. Hamadani et al. (2014) did not detect basophils in adult domestic geese [8], whereas low basophil percentages were reported in adult greylag geese [9]. The basophil percentage reported in adult male Chinese geese by Benarrós et al. (2020) was numerically higher than the values reported in these two studies [7]. These differing findings suggest that circulating eosinophil and basophil percentages may vary among goose populations. However, because parasitological examinations and inflammatory markers were not evaluated, the available data do not permit a definitive conclusion as to whether the observed eosinophil differences were associated with an inflammatory, parasitic, or other pathological process.

The effect sizes were evaluated according to leukocyte parameters (Table 2); it was found that the total leukocyte count, lymphocyte percentage, heterophil percentage, and H/L ratio varied depending on age and the age × sex interaction. The effect of sex on the variation in these parameters was less significant. For the monocyte and eosinophil percentages, age, sex, and their interactions had a significant effect on the results. Although the effect size values for the basophil percentage indicated that age had a more significant effect on the results, ANOVA results indicated that neither of these parameters had a significant effect on the variation in this value.

In the present study, TAS values were higher in 98-week-old geese than in 16-week-old geese, while TOS and OSI did not differ between the age groups. TAS is a composite measure of the total antioxidant capacity of a biological sample; however, it does not indicate the extent to which individual antioxidant components contribute to the measured total value [31]. In a study of songbird species living in the wild, it was reported that the relationship between non-enzymatic antioxidant capacity in plasma and reactive oxygen metabolites varied depending on the year and experimental conditions [38]. These findings suggest that antioxidant capacity and other oxidation-related indicators may not change in the same direction under all conditions. Consequently, elevated TAS levels alone do not necessarily indicate a more robust antioxidant defense, reduced oxidative stress, or diminished oxidative damage [31,38].

Regarding sex-related differences, TOS and OSI values were higher in female geese than in male geese, while TAS did not differ between the sexes. TOS provides information about the total oxidant status of a sample [30], while OSI is based on the ratio of TOS to TAS [32]. Accordingly, the higher OSI in females occurred alongside higher TOS without a significant sex-related difference in TAS. This combination of findings is consistent with the calculation of OSI. Yavuz et al. (2023) reported that TOS differed significantly among live-weight categories in nine-month-old clinically healthy geese, but the effect of sex on TOS was not significant [22]. Nevertheless, mean TOS was numerically higher in females, as observed in the present study. In the same study, ferric reducing antioxidant power (FRAP) was significantly higher in females; TAS, OSI, and GSH showed no significant sex-related differences [22]. Direct comparison is limited because their study evaluated live-weight categories within a single age group of nine-month-old geese, while the present study compared geese aged 16 and 98 weeks. Taken together, the available findings indicate that sex-related differences do not occur uniformly across all components of oxidant–antioxidant status. The age × sex interaction did not yield significant effect sizes for the oxidant–antioxidant status parameters. Meanwhile, particularly the age had a high effect size for TAS. However, for TOS and OSI, sex had a high effect size.

The effects of dietary composition and nutritional supplements on blood parameters in geese have been reported [39,40]. In this study, all geese were fed the same diet from 10 weeks. Feeding all geese the same grower diet standardized the composition of the feed offered across the groups. However, the use of the same diet does not establish that individual feed intake was comparable between 16- and 98-week-old geese or that their age-specific nutrient requirements were met to the same extent. In 10-month-old male geese kept under warm environmental conditions, dietary L-arginine supplementation affected erythrocyte count, hemoglobin concentration, PCV, total leukocyte count, and differential leukocyte values but did not affect MCV or MCHC [39]. In young Tetra-SL chicks, methionine source and level affected erythrocyte and leukocyte counts, hemoglobin concentration, and hematocrit but had no significant effect on MCV, MCH, or MCHC [41]. In a study, it was reported that different levels of Azolla in the diet altered the SOD, GSH, and CAT levels, which are oxidant–antioxidant parameters, in growing geese, but did not affect MDA levels [40]. These studies indicate that diet-related effects do not occur uniformly across all hematological and oxidant–antioxidant parameters. However, these intervention studies do not demonstrate that the differences observed between the age groups in the present study resulted from the use of the same grower diet. Although the uniform diet standardized the feed offered, the potential contribution of individual feed intake or digestive efficiency to the observed differences could not be isolated under the current experimental design.

The interpretation of these findings is best considered within the specific scope of this study. Because only two discrete age groups, 16 and 98 weeks, were compared, the results are limited to differences between these groups. The study design does not allow the course of change between these ages to be characterized or the observed differences to be attributed to maturation or processes occurring later in life. As discussed above, all geese received the same grower diet, and individual feed intake was not assessed; therefore, whether or to what extent diet contributed to the differences between the two age groups could not be determined. The wide confidence intervals around several effect-size estimates also indicate uncertainty regarding the magnitude of the observed effects. Future studies incorporating intermediate age groups or longitudinal designs with repeated measurements, age-appropriate feeding programs with individual feed-intake monitoring, and direct markers of oxidative damage could provide a more detailed evaluation of hematological and oxidant–antioxidant parameters.

## 5. Conclusions

In conclusion, significant age × sex interactions were detected for erythrocyte count, PCV, MCHC, and all evaluated leukocyte parameters except basophil percentage in clinically healthy native Turkish white geese aged 16 and 98 weeks. No significant effects of age, sex, or the age × sex interactions were detected for hemoglobin concentration, MCV, MCH, or basophil percentage. No significant age × sex interaction was detected for TAS, TOS, or OSI. TAS values were higher in 98-week-old geese than in 16-week-old geese, while TOS and OSI values were higher in female geese than in male geese. These comparative data may provide supplementary information for veterinarians and researchers when interpreting hematological and oxidant–antioxidant measurements in clinically healthy native Turkish white geese within these age groups and under similar rearing conditions. However, the findings are limited to the two distinct age groups and study conditions examined. They do not characterize changes throughout the lifespan of geese, identify the physiological mechanisms underlying the observed differences, or provide evidence of differences in oxidative damage or health status.

## Figures and Tables

**Table 1:** Hematological parameters and oxidant–antioxidant status of native Turkish white geese.

Parameters	Young Female	Adult Female	Young Male	Adult Male	*p* (Age)	*p* (Sex)	*p* (Interaction)
Erythrocyte parameters							
Erythrocyte count (×10^6^/mm^3^)	2.15 ± 0.34 ^ab^	2.10 ± 0.27 ^ab^	2.40 ± 0.29 ^a^	1.99 ± 0.26 ^b^	0.001 ***	0.375	0.000 ***
Packed Cell Volume (%)	42.91 ± 3.02 ^a^	37.93 ± 2.85 ^b^	44.24 ± 6.56 ^a^	36.81 ± 2.98 ^b^	0.000 ***	0.918	0.000 ***
Hemoglobin (g/dL)	18.15 ± 3.48	16.93 ± 1.72	17.21 ± 4.24	16.06 ± 2.67	0.062	0.157	0.138
MCV (fL)	206.16 ± 40.90	182.05 ± 19.41	190.17 ± 35.77	187.27 ± 20.39	0.070	0.473	0.118
MCH (pg)	87.19 ± 26.46	81.20 ± 9.69	73.38 ± 21.53	80.99 ± 13.19	0.831	0.064	0.080
MCHC (g/dL)	42.18 ± 6.72 ^ab^	44.59 ± 4.42 ^a^	39.02 ± 6.27 ^b^	43.62 ± 4.54 ^a^	0.009 **	0.129	0.019 *
Leukocyte parameters							
Total leukocyte count (×10^3^/mm^3^)	6.69 ± 2.34 ^b^	8.37 ± 1.34 ^a^	6.78 ± 1.41 ^b^	7.96 ± 1.52 ^a^	0.000 ***	0.666	0.001 ***
Lymphocyte (%)	61.45 ± 4.62 ^a^	46.45 ± 12.55 ^b^	60.64 ± 3.66 ^a^	45.82 ± 6.49 ^b^	0.000 ***	0.767	0.000 ***
Heterophil (%)	33.64 ± 4.73 ^b^	47.73 ± 12.25 ^a^	33.91 ± 4.17 ^b^	49.00 ± 7.70 ^a^	0.000 ***	0.756	0.000 ***
Monocyte (%)	2.00 ± 0.82 ^a^	1.45 ± 0.68 ^b^	1.73 ± 0.52 ^ab^	0.82 ± 0.42 ^c^	0.000 ***	0.003 **	0.000 ***
Eosinophil (%)	1.18 ± 0.42 ^c^	2.00 ± 0.82 ^ab^	1.64 ± 0.74 ^bc^	2.27 ± 0.95 ^a^	0.000 ***	0.038 *	0.000 ***
Basophil (%)	1.73 ± 1.06	2.36 ± 0.79	2.09 ± 1.84	2.09 ± 2.23	0.315	0.886	0.566
H/L ratio	0.56 ± 0.12 ^b^	1.04 ± 0.47 ^a^	0.72 ± 0.16 ^b^	1.11 ± 0.37 ^a^	0.000 ***	0.210	0.000 ***
Oxidant–antioxidant status parameters							
TAS (mmol Trolox equivalent/L)	1.13 ± 0.083	1.22 ± 0.130	1.21 ± 0.102	1.23 ± 0.118	0.024 *	0.062	0.170
TOS (µmol H_2_O_2_ equivalent/L)	16.18 ± 7.66	14.59 ± 6.98	12.13 ± 6.38	11.10 ± 7.56	0.376	0.009 **	0.303
OSI (AU)	1.45 ± 0.34	1.18 ± 0.27	1.02 ± 0.25	0.90 ± 0.20	0.123	0.004 **	0.142

a, b, c within each row, different superscript letters indicate a significant difference between age × sex interactions. All *p*-values were calculated using ANOVA and Bonferroni-adjusted tests. (*): *p* < 0.05; (**): *p* < 0.01; (***): *p* < 0.001.

**Table 2:** Partial eta-squared (ηp^2^) effect sizes and 95% confidence intervals for the effects of age, sex and age × sex interaction on hematological parameters and oxidant–antioxidant status in geese.

Parameters	Age ηp^2^ (95% CI)	Sex ηp^2^ (95% CI)	Age × Sex ηp^2^ (95% CI)
Erythrocyte parameters			
Erythrocyte count (×10^6^/mm^3^)	0.263 (0.054–0.474)	0.022 (0.000–0.186)	0.372 (0.134–0.564)
Packed Cell Volume (%)	0.413 (0.172–0.596)	0.001 (0.000–0.091)	0.298 (0.077–0.504)
Hemoglobin (g/dL)	0.093 (0.000–0.299)	0.055 (0.000–0.246)	0.060 (0.000–0.254)
MCV (fL)	0.088 (0.000–0.293)	0.014 (0.000–0.165)	0.067 (0.000–0.264)
MCH (pg)	0.001 (0.000–0.091)	0.092 (0.000–0.298)	0.083 (0.000–0.286)
MCHC (g/dL)	0.175 (0.013–0.391)	0.063 (0.000–0.258)	0.144 (0.003–0.359)
Leukocyte parameters			
Total leukocyte count (×10^3^/mm^3^)	0.313 (0.087–0.516)	0.005 (0.000–0.132)	0.263 (0.054–0.474)
Lymphocyte (%)	0.513 (0.277–0.670)	0.002 (0.000–0.108)	0.512 (0.276–0.669)
Heterophil (%)	0.487 (0.248–0.651)	0.003 (0.000–0.118)	0.423 (0.181–0.603)
Monocyte (%)	0.433 (0.191–0.611)	0.220 (0.031–0.435)	0.347 (0.113–0.544)
Eosinophil (%)	0.297 (0.076–0.503)	0.114 (0.000–0.325)	0.301 (0.079–0.506)
Basophil (%)	0.028 (0.000–0.198)	0.001 (0.000–0.091)	0.009 (0.000–0.149)
H/L ratio	0.346 (0.113–0.543)	0.043 (0.000–0.226)	0.392 (0.152–0.580)
Oxidant–antioxidant status parameters			
TAS (mmol Trolox equivalent/L)	0.134 (0.001–0.348)	0.093 (0.000–0.299)	0.052 (0.000–0.241)
TOS (µmol H_2_O_2_ equivalent/L)	0.022 (0.000–0.186)	0.175 (0.013–0.391)	0.029 (0.000–0.200)
OSI (AU)	0.065 (0.000–0.261)	0.208 (0.026–0.423)	0.059 (0.000–0.252)

## Data Availability

The original contributions presented in this study are included in the article. Further inquiries can be directed to the corresponding author.
